# Data set in support of neurotoxicity of trimethyltin chloride by morphological and protein analysis

**DOI:** 10.1016/j.dib.2016.01.021

**Published:** 2016-01-16

**Authors:** C-Yoon Kim, Jin Kim, Juha Song, Hanseul Oh, Jae-Hak Park

**Affiliations:** Laboratory Animal Medicine, College of Veterinary Medicine and Research Institute for Veterinary Science, Seoul National University, Seoul 151-742, Republic of Korea

**Keywords:** Trimethyltin chloride, Neurotoxicity, Zebrafish

## Abstract

Trimethyltin chloride (TMT) is a neurotoxicant widely present in the aquatic environment. Chronic exposure of embryos to TMT for 4 days post-fertilization (dpf) elicited a concentration-related decrease in head & eye size and increase in axial malformation. In addition, Rohon-Beard sensory neurons and motor neurons showed decreased patterns of protein expression. These data coincide with previous research about the neurotoxicity of TMT on mRNA expression (Kim et al., 2016 [1]). These data demonstrates that TMT inhibits specific neurodevelopmental stages in zebrafish embryos and suggests a possible mechanism for the toxicity of TMT in vertebrate neurodevelopment. This paper contains data related to research concurrently published in Kim et al. (2016) [1].

**Specifications Table**

**Table:** 

Subject area	*Biology*
More specific subject area	*Neurotoxicology*
Type of data	*Figure, graph*
How data was acquired	*Immunohistochemistry, fluorescence microscope, stereoscope and real time PCR (qPCR)*
Data format	*Raw, analyzed*
Experimental factors	*Chronic exposure of zebrafish embryos to Trimethyltin chloride*
Experimental features	*Developmental abnormalities in zebrafish*
Data source location	*Seoul National University, Seoul, South Korea*
Data accessibility	*Data is provided within the article*

**Value of the data**

• These data show that chronic exposure of TMT elicit neurotoxicity in developmental stage in zebrafish.
• These data show the dose-dependent effects of TMT on morphological defects in the head formation of zebrafish.
• These data show the specific neurotoxicity of TMT on neurodevelopmental stage in zebrafish.

## 1. Data

**Fig. 1:**
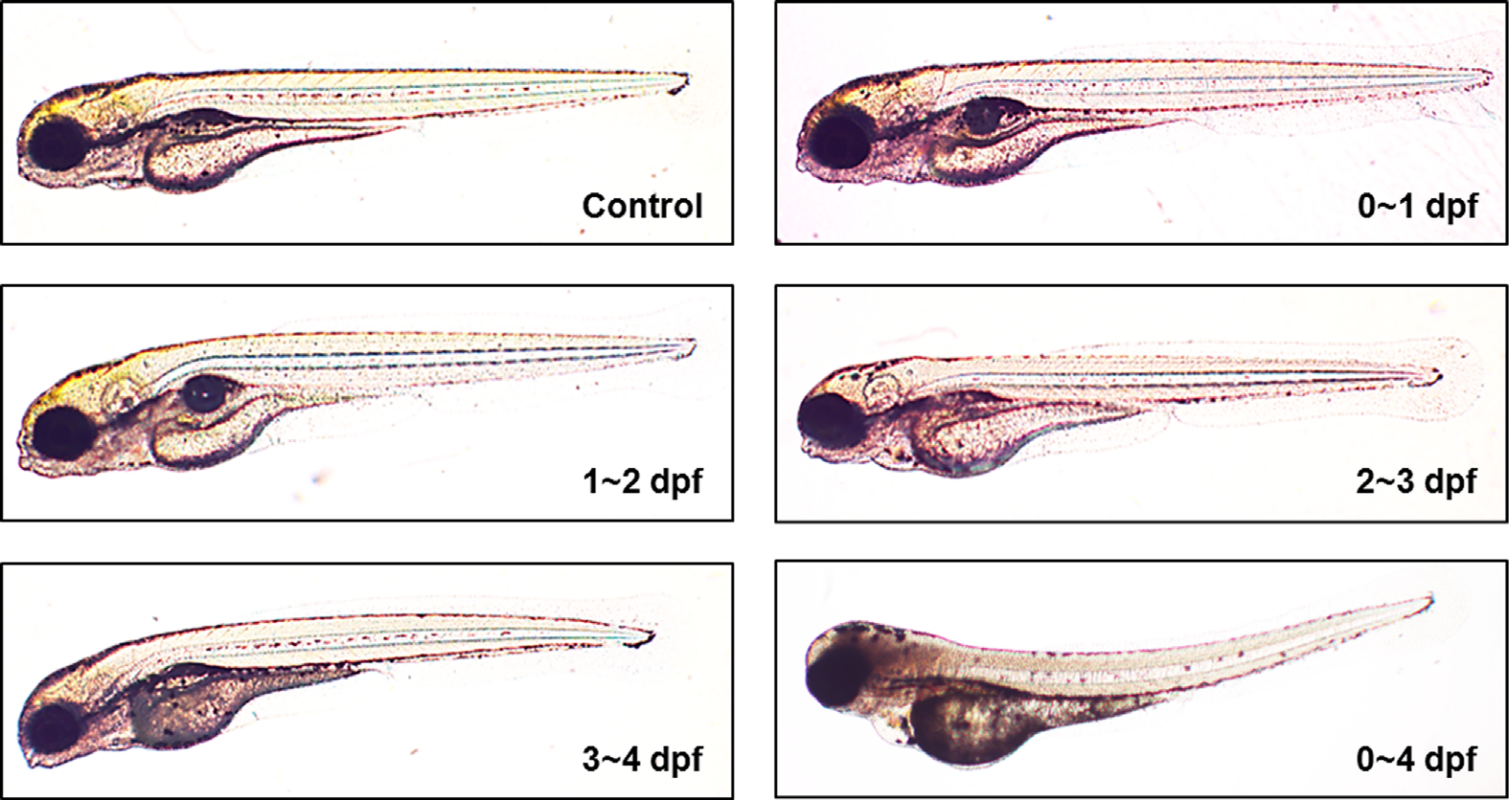
Acute/chronic effect of TMT exposure on zebrafish embryo. Normal morphology of control and acute exposure groups (0–1; 1–2; 2–3 and 3–4 dpf) and abnormal morphology induced by chronic exposure (0–4 dpf).

**Fig. 2:**
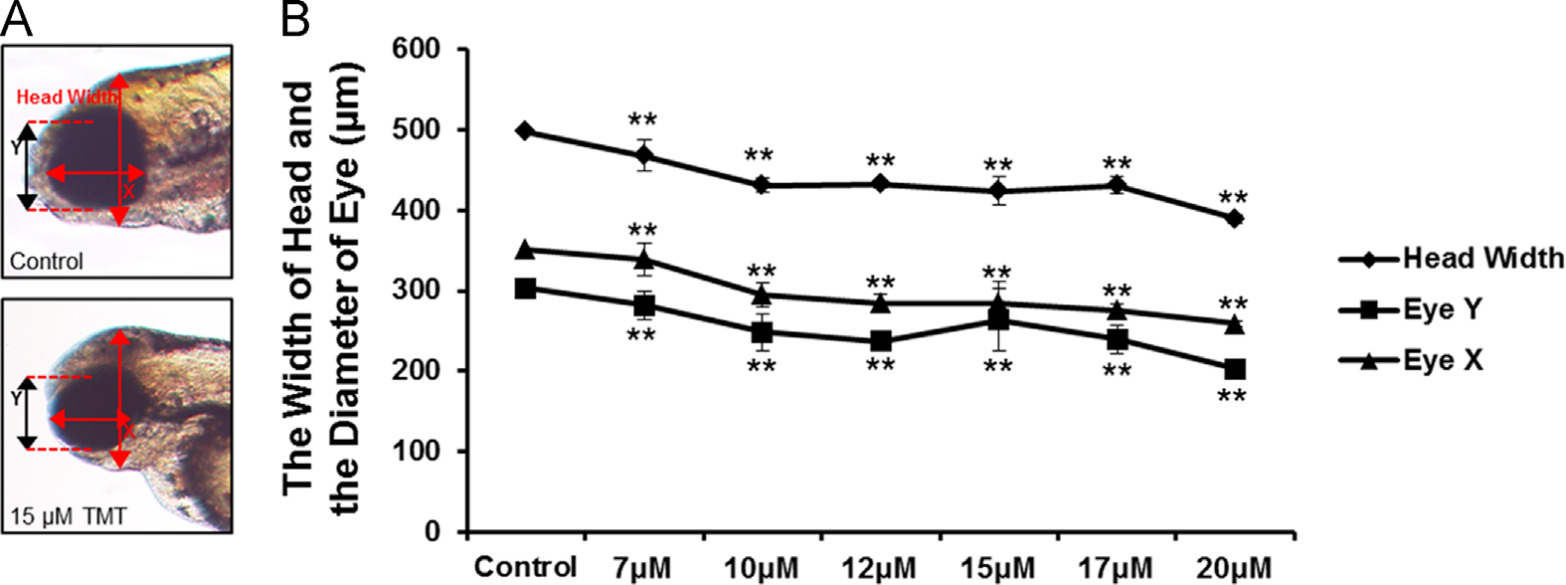
TMT-induced morphological defects in head & eye size. The measurement of head & eye size (A). The significant decrease of head & eye size in a dose dependent manner (B). Data represent the mean±SD of three experiments. **P<0.01.

**Fig. 3:**
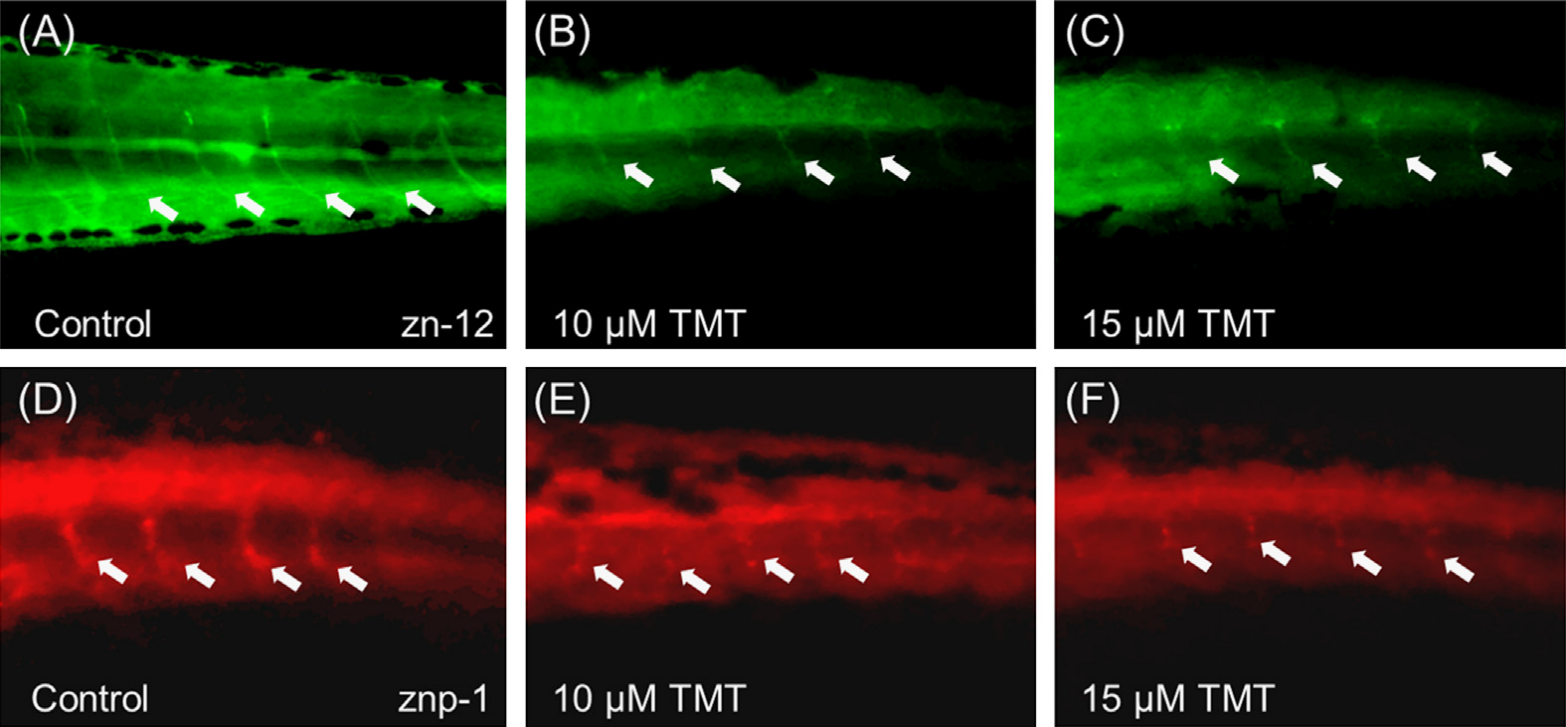
TMT-induced neurotoxicity on sensory and motor neuron development. Zn-12 (green) labeling showing Rohon-Beard sensory neurons (A–C) and znp-1 labeling (red) showing motor axons (D–F). The arrows indicate the neuron axon.

Neurotoxicity of TMT acute exposure was not detected in early developmental stage. However, prolonged exposure of TMT induced abnormal morphology in zebrafish embryo (Fig. 1). In addition, chronic exposure of TMT elicited morphological defects in head including head & eye size in a dose dependent manner (Fig. 2A, B). To evaluate the specific neurotoxicity, immunohistochemistry was performed on Rohon-Beard sensory neurons and motor neuron, which confirmed specific neurodevelopmental inhibition induced by TMT. At 10 μM, morphological differences in Rohon-Beard sensory neurons (zn-12) and motor neuron (znp-1) positive neurons were noted in ventrally projecting axons compared with the control group (Fig. 3A, B, D and E). Moreover, it was difficult to detect morphologic normality in axons or axon branches at 15 μM (Fig. 3C, F). This paper contains data related to research concurrently published in [1].

## 2. Experimental design, materials and methods

### 2.1 Chemical exposure on zebrafish embryos

Fertilized zebrafish embryos were obtained from healthy male and female zebrafish. Embryos were collected and washed with zebrafish embryo medium. Trimethyltin chloride (Sigma-Aldrich, MO, USA) was purchased from Sigma-Aldrich. TMT concentration were 0 (embryo medium), 5, 7, 10, 15, 17, and 20 μM. TMT was dissolved in embryo medium.

### 2.2 Embryo–larvae Toxicity assay

All the procedures using zebrafish embryos were approved from the Institutional Animal Care and Use Committee of Seoul National University. Embryos and larvae were monitored for morphological analysis at every 0.5 dpf for 4 days using an Olympus IX70 (Olympus, Japan) at 4 dpf.

### 2.3 Whole-mount immunohistochemistry

Whole-mount immunohistochemistry was performed according to McGraw et al. [2] with some modifications. Briefly, embryos were fixed overnight in 4% formaldehyde in phosphate-buffered saline (PBS). Fixed embryos were washed with in PBS with 0.1% Triton X-100 (PBT), then treated for 20 min at −20 °C with acetone. For staining, embryos were incubated with mouse anti-zn-12 (1:500; ZIRC, OR, USA) and mouse anti-znp-1 (1:1000; ZIRC) for 3 days at 4 °C. After extensive washing with PBT, embryos were incubated in Alexa-488- or Alexa-594-conjugated secondary antibodies (Invitrogen) for 2 days at 4 °C, rinsed in PBT, and then mounted in 50% glycerol/PBS for imaging.

### 2.4 Statistical analysis

A one‐way analysis of variance (ANOVA) followed by the Tukey HSD test was performed in SPSS Statistics (version 16). Experimental data were checked to determine if there was a significant difference. The level for statistical significance was set at the 0.05 and 0.01.
