# Supplementary figures and images for: Data set in support of neurotoxicity of trimethyltin chloride by morphological and protein analysis
Source: Data Brief. 2016 Jan 16;6:706–9. doi: 10.1016/j.dib.2016.01.021 (PMC4773414; doi:10.1016/j.dib.2016.01.021)

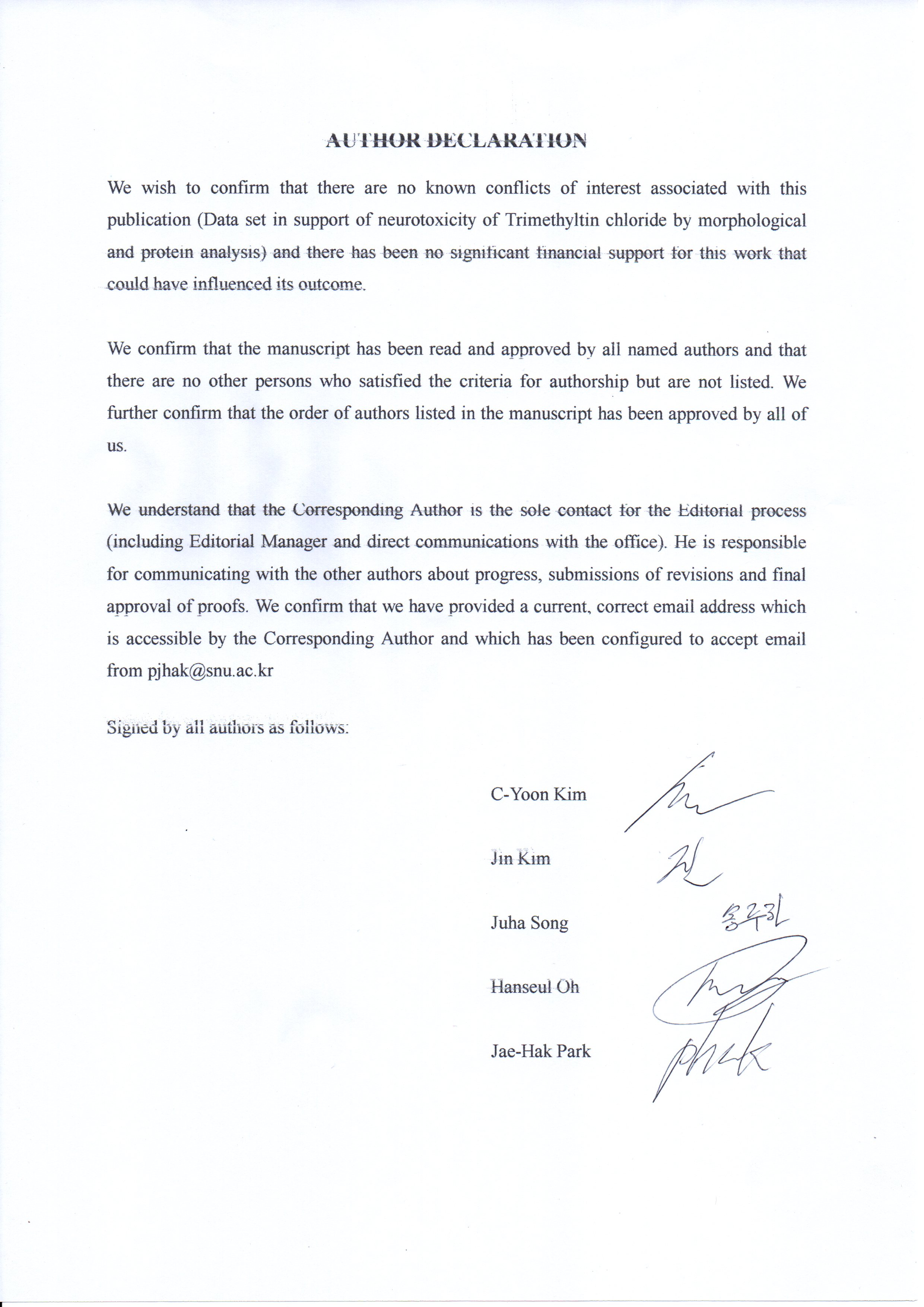

Supplement: Supplementary file 1 — Supplementary material [file mmc1.zip › Athor declaration_C.jpg]
